# The mediating role of negative emotions in the relationship between smoking and health-related quality of life among Chinese individuals: A cross-sectional study

**DOI:** 10.18332/tid/171355

**Published:** 2023-10-16

**Authors:** Zhenni Luo, Weihong Xu, Shijing Jiang, Qian Zhou, Yan Guan, Lu Li, Siyuan Liu, Haozheng Zhou, Xuanhao Yin, Yibo Wu, Jiangyun Chen

**Affiliations:** 1 School of Health Management, Guangzhou Medical University, Guangzhou, China; 2 School of Health Management, Southern Medical University, Guangzhou, China; 3 School of Public Health, Peking University, Beijing, China; 4 Center for WHO Studies, Department of Health Management, School of Health Management of Southern Medical University, Guangzhou, China

**Keywords:** smoking, negative emotion, health-related quality of life, depression, anxiety

## Abstract

**INTRODUCTION:**

Although the negative impact of smoking on health has been confirmed in various studies, few have explored psychological factors mediating the relationship between smoking and health-related quality of life (HRQOL). This study aimed to investigate the relationship between smoking and HRQOL in the Chinese population and the mediating role of negative emotions (NEs).

**METHODS:**

Survey data were derived from a cross-sectional study conducted in China from 20 June to 31 August 2022. We recruited participants from 148 cities across the country using a stratified multistage sampling method. The HRQOL of the dependent variable was measured using the Chinese version of European Quality of Life-5 Dimensions (EQ-5D-5L). The Patient Health Questionnaire (PHQ-9), Generalized Anxiety Disorder (GAD-7), and Perceived Stress Scale (PSS-4) were used to measure NE parameters including depression, anxiety, and perceived stress, as the intermediate variables. A multiple parallel mediation model was used to analyze the mediating role of NEs in smoking and HRQOL.

**RESULTS:**

A total of 21916 valid questionnaires were collected, of which 3010 (13.7%) and 18906 (86.3%) were categorized into smokers and non-smokers, respectively. The HRQOL (EQ-VAS score) of smokers (71.70 ± 23.08) was lower than that of non-smokers (73.69 ± 21.32), whereas the depression and anxiety levels of smokers were higher than those of non-smokers (all p<0.001). Moreover, smoking, NEs (depression and anxiety), and HRQOL showed pairwise correlations. According to the mediation analysis, depression (β= -0.461; 95% BCa CI: -0.664 – -0.268) and anxiety (β= -0.279; 95% BCa CI: -0.435 – -0.138) mediated the relationship between smoking and HRQOL after adjusting for demographic and life factors.

**CONCLUSIONS:**

These findings emphasize the necessity of studying the interaction between smoking, HRQOL, and Nes, and complementing the research on the impact of psychological factors on the HRQOL of smokers. Public health activities should focus on mental health and take targeted measures for the prevention, treatment, and rehabilitation of smokers.

## INTRODUCTION

Tobacco use is one of the most serious public health problems worldwide. Smoking caused approximately 100 million deaths globally in the 20th century, mostly in low- and middle-income countries, including China^1^. China is the largest producer and consumer of tobacco, consuming about 40% of the world’s tobacco; the total number of smokers in China exceeds 300 million, accounting for one-third of the world’s smoking population^2^. China’s economy, social order, and public health have suffered tremendously as a result of heavy tobacco consumption, with approximately one million deaths per year from tobacco-related diseases^3^.

In addition to the direct harmful consequences to an individual’s survival, smoking also impacts health-related quality of life (HRQOL)^4^, a multidimensional evaluation index that includes physical health, psychological status, social relationships, and subjective satisfaction. It facilitates not only the comprehensive evaluation of an individual’s health status but also the policy legislation and health plan implementation for health institutions. Previous studies have shown a significant negative association between smoking and HRQOL, with non-smokers or former smokers having higher HRQOL scores than long-term smokers^5^. The association between smoking and HRQOL in terms of physical factors has been extensively examined, whereas the discussion surrounding psychological factors in this relationship has been comparatively limited. In addition, smoking is reported to have a stronger correlation with the mental health part than the physical health part of HRQOL^5^, possibly due to the worsening of negative emotions (NEs)^6^. Accordingly, it is of great significance to clarify the complex path relationship between the smoking and HRQOL.

NEs are subjective experiences of depression and unpleasantness that include various emotional states^7^. These not only lead to increases in problematic behavior but are also important causes of emotional disorders^8^. Although NEs are often reported as factors that induce smoking, an interaction between these two parameters has also been observed^9^.

NE is an important risk factor for HRQOL^10^. Further, the emotional states of depression, anxiety, and stress are closely related, together comprising NE^11^. Smoking is a reported risk factor for depression, anxiety, and stress. Compared to non-smokers, smokers have more serious symptoms of depression and anxiety^12^. Therefore, further studies are necessary to determine whether the components of NE mediate the relationship between smoking and HRQOL.

Few studies have elaborated on the interaction between smoking and HRQOL in low- and middle-income countries. Although China is the world’s largest producer and consumer of tobacco, far fewer studies on the effects and mechanisms linking smoking and HRQOL have been published in China than those in Western countries^13^. Furthermore, previous studies have only discussed the relationship between smoking and HRQOL in specific populations in terms of disease, lifestyle, and demographic factors^14,15^, but studies on the general population are lacking. In addition, most studies have just explored the effects of individual emotions, such as depression, on smoking and HRQOL^16^, and have not fully explored the overall impact of a group of NEs. Thus, the associations between smoking, NEs, and HRQOL should be investigated in the general Chinese population.

**Figure 1:**
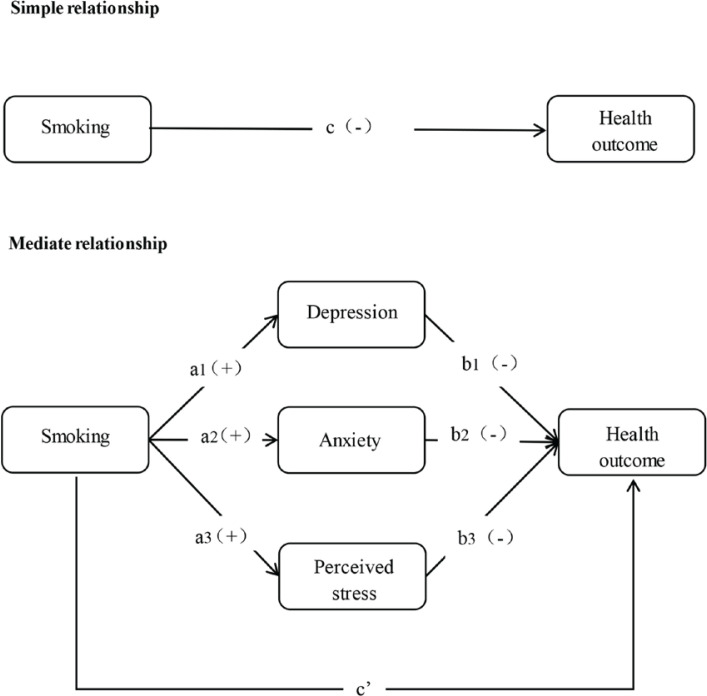
Multiple parallel mediating hypothesis model of smoking, negative emotions (NEs), and healthrelated quality of life (HRQOL)

This study aimed to investigate the association between smoking and HRQOL in the Chinese population and to determine the mediating role of NEs (depression, anxiety, and perceived stress) using a multiple mediation model (Figure 1). Based on previous findings, we hypothesized that: 1) smoking is negatively correlated with HRQOL; and 2) the three NE variables play an intermediary role in the relationship between smoking and HRQOL.

## METHODS

### Participants and survey design

The data for this study were obtained from a cross-sectional survey of Chinese residents. The survey was conducted from 20 June to 31 August 2022 involving 23 provinces, 5 autonomous regions, and 4 municipalities in mainland China. In total, 148 cities were extracted using a random number table. From each province or autonomous region, we included the provincial capitals and 2–6 cities of different sizes. In total, 202 districts, 390 towns/streets, and 780 communities/villages were selected using stratified and quota sampling. Potential participants were contacted via phone or face-to-face by investigators. To ensure the effective implementation of the survey, investigators were publicly recruited in each city and strictly trained, including standardized processes and anticipating unforeseen circumstances, to respond to the survey. The online questionnaire was implemented using Wenjuanxing (a professional online questionnaire survey platform, https://www.wjx.cn/) and shared with participants in face-to-face sessions.

In total, 23414 questionnaire responses were collected. After identifying and deleting duplicate values, missing values, and outliers with logical problems, 21916 samples were obtained, an effective response rate of 93.6%. The inclusion criteria were as follows: 1) age ≥12 years, 2) Chinese nationality, 3) Chinese residence (annual travel time ≤1 month), 4) voluntary participation in the study, with informed consent, 5) completion of the online questionnaire survey either alone or with the help of the trained investigators; and 6) comprehension of each item in the questionnaire. Exclusion criteria were: 1) participants who were diagnosed or self-reported with mental disorders, 2) cognitive impairment; and 3) participation in other similar research topics.

Ethical approval was obtained from the Ethics Committee of Peking University. Before participation, the investigators clearly stated the purpose and content of the study. After obtaining informed consent, the participants were able to answer the questionnaire and had the right to withdraw their consent at any time. The data of all participants were anonymized for further analysis.

### Measurements

*Health*

HRQOL in this study was measured using the Chinese version of European Quality of Life-5 Dimensions (EQ-5D-5L) questionnaire^17^, which consists of two parts: a health questionnaire and utility conversion value. The health questionnaire consists of the EQ-5D descriptive system and the European Quality-Visual Analogue Scale (EQ-VAS). The EQ-5D descriptive system comprises five dimensions: mobility, self-care, usual activities, pain/discomfort, and anxiety/depression^18^. The EQ-VAS is a standard, vertical, 20 cm visual analogue scale. The scale ranges from 100 (the best health state one can imagine) to 0 (the worst health one can imagine)^19^. Cronbach’s coefficient for this scale in this study was 0.624.

The intermediate variables in this study include anxiety and depression, partially overlapping with the five dimensions of the descriptive EQ-5D system. The EQ-5D health index converts the five-dimensional measurements into a single index value and uses it as a dependent variable. Although both the EQ-5D health index and EQ-VAS are measures of overall health, the former estimates the value of a respondent’s health from the perspective of the general public, whereas the latter takes the perspective of the respondent^14,17^. Therefore, this study used the EQ-VAS score as the dependent variable, which can directly obtain information on the subjective perception of a participant’s health status at that time.

### Smoking

Participants were asked: ‘Do you have a habit of smoking?’. Response options were: ‘yes’, ‘have quit smoking’ or ‘no’. Based on this question, smoking status was dummy-coded as: 1) smoking (participants who smoked); or 2) non-smoking (participants who had never smoked or had quit smoking).

### Negative emotions

In a study of NEs and quality of life in Chinese adolescents, Geng et al.^20^ demonstrated that depression, anxiety and perceived stress combined, are associated with lower quality of life. To examine the magnitude of the mediating effects of depression, anxiety, and stress on NEs, the following three separate scales were used for testing.

*Perceived stress*

The Perceived Stress Scale (PSS)-4 was developed by Cohen et al.^21^ to measure an individual’s perceived stress, ability to cope with it, and related coping characteristics. It is rated on a 5-point Likert scale from 0 to 4 for each item, ranging from ‘never’ to ‘always’, with questions 1 and 4 being positively assigned and questions 2 and 3 being negatively assigned. A higher total score on the scale indicates a higher perception of stress. Cronbach’s α coefficient for this scale in this study was 0.826.

*Anxiety*

The Generalized Anxiety Disorder (GAD)-7 is a brief self-rating scale of anxiety symptoms developed by Spitzer et al.^22^, based on the Diagnostic and Statistical Manual of Mental Disorders, 4th edition (DSM-IV) diagnostic criteria in the United States. GAD-7 has seven items, each rated on a 4-point scale, from 0 to 3, with the total score being the main statistical indicator. A higher score corroborated with more severe anxiety. A total score of 0–4 indicates no clinically significant anxiety, 5–9 indicates mild anxiety, 10–14 indicates moderate anxiety, and ≥15 indicates severe anxiety. The maximum sensitivity and specificity were obtained when a score of 10 was obtained. Cronbach’s α coefficient for this scale in this study was 0.942.

*Depression*

The Patient Health Questionnaire (PHQ)-9 is a brief and validated self-assessment scale of depressive status based on the nine items of the DSM-IV diagnostic criteria and is used to rate the severity of the respondent’s depressive status. It consists of nine items, with each item’s response consisting of four options: ‘not at all’, ‘a few days’, ‘more than half of the days’, and ‘almost every day’, with scores of 0, 1, 2, and 3, respectively. The maximum total score is 27. A total score of ≥5 on the assessment was considered positive, indicating the possible presence of depressive mood, with scores of 5–9 deemed likely to have mild depression, 10–14 likely to have moderate depression, and 15–27 likely to have severe depression^23^. In the present study, Cronbach’s α coefficient for this scale was 0.921.

### Sociodemographic variables

Factors potentially influencing HRQOL were identified from the literature^5,14^. The demographic characteristics included sex, age, residence (urban, rural), occupational status (student, work, and retired/unemployed), education level (primary school and below, junior high school, senior high school, and undergraduate and above), and monthly household income (≤2000; 2001–5000; and ≥5001 RMB). Lifestyle factors incorporated drinking status (whether or not alcohol was consumed) and the presence or absence of self-reported chronic conditions, including hypertension, coronary heart disease, and stroke.

### Statistical analysis

All statistical analyses were performed using IBM SPSS Statistics version 24 (IBM Corp., Armonk, NY, USA). All statistical tests were two tailed. Data normality was assessed using the Kolmogorov–Smirnov normality test. Statistical descriptive data included mean and standard deviation, and frequency and percentage. The t-test was used for continuous variables, and the chi-squared test or Kruskal–Wallis one-way ANOVA were used for categorical variables to compare the differences between smokers and non-smokers. In the subsequent hypothesis test, collinearity test results were first used to ensure that the expansion factor of the variable did not exceed 10, indicating no collinearity between variables. Moreover, the Harman single-factor test was used to assess the common method bias. The results showed that the first factor without rotation was 35.8%, which is less than the standard boundary value of 40%, suggesting no serious common method bias^24^. Pearson’s correlation coefficient and the eta (η) coefficient were used to evaluate correlations between HRQOL, NEs (depression, anxiety, and stress), and smoking status.

We used a multiple parallel mediation model to assess the effect of smoking (binary variable) on HRQOL (continuous variable), and the mediating role of NE (continuous variable). Because the independent variable was binary, the relative mediation analysis method proposed by Hayes and Preacher was used^25^. The relative mediating effect was further tested by bootstrap repeated sampling using the PROCESS macro of SPSS. The bootstrap test resamples the population data with replacements for the same number of *n* data points, and this procedure is repeated for *k* samples. The recommended sampling size is *k*=5000. Bias-corrected and accelerated bootstrap confidence intervals (95% BCa CIs) were used to correct for bias and skewness. It was considered statistically significant if the confidence interval did not include the value 0 for the indirect effect of smoking on HRQOL^16^. Finally, we obtained the total, direct, and indirect mediating effects of each variable. We established five equations to estimate the impact of smoking on HRQOL:

*Y* = *i* + *cX* + *e*

*M*_1_ *= i M*_1_ *+ a*_1_ *X + eM*_1_

*M*_2_ *= i M*_2_ *+ a*_2_ *X + eM*_2_

*M*_3_ *= i M*_3_ *+ a*_3_ *X + eM*_3_

*Y = iY + c’X + b*_1_ *M*_1_ *+ b*_2_ *M*_2_ *+ b*_3_ *M*_3_ *+ eY*

where *Y* represents HRQOL, *X* smoking, *M*_1_ depression, *M*_2_ anxiety, *M*_3_ perceived stress, *c* the total effect of *X* on *Y, c’* the direct effect of *X* on *Y, a*_i_ the effect of *X* on *M*_i_ (*i* = 1 to 3), *b*_i_ the total effect of *M*_i_ on *Y* (*i* = 1 to 3), *I* the intercept, and *e* the error.

## RESULTS

### Characteristics of the participants

Table 1 shows the basic characteristics of the 21916 participants. The total sample was balanced between men and women (50.0% each), with 15188 (69.3%) urban and 6728 (30.7%) rural participants, and an average age of 39.43 ± 18.85 years, making the sample representative.

**Table 1:** The characteristics of the study participants in China, June–August 2022 (N=21916)

*Characteristics*	*All (N=21916) n (%)*	*Smoking (N=3010) n (%)*	*Non-smoking (N=18906) n (%)*	*p*
**Sex**				<0.001
Male	10958 (50.0)	2711 (90.1)	8247 (43.6)	
Female	10958 (50.0)	299 (9.9)	10659 (56.4)	
**Age** (years), mean ± SD	39.43 ± 18.85	44.29 ± 16.83	38.65 ± 19.04	<0.001
**Employment status**				<0.001
Student	6580 (30.0)	393 (13.1)	6187 (32.7)	
Employed	7601 (34.7)	1330 (44.2)	6271 (33.2)	
Retired/unemployed	7735 (35.3)	1287 (42.8)	6448 (34.1)	
**Residence** (last three months)				<0.001
Urban	15188 (69.3)	1959 (65.1)	13229 (70.0)	
Rural	6728 (30.7)	1051 (34.9)	5677 (30.0)	
**Education level**				<0.001
Primary school and lower	3412 (15.6)	538 (17.9)	2874 (15.2)	
Junior high school	4739 (21.6)	808 (26.8)	3931 (20.8)	
Senior high school	6512 (29.7)	953 (31.7)	5559 (29.4)	
Undergraduate and higher	7253 (33.1)	711 (23.6)	6542 (34.6)	
**Monthly household income** (RMB)				<0.001
≤2000	3847 (17.6)	618 (20.5)	3229 (17.1)	
2001–5000	10037 (45.8)	1309 (43.5)	8728 (46.2)	
≥5001	8032 (36.6)	1083 (36.0)	6949 (36.8)	
**Chronic**				0.009
No	16412 (74.9)	2196 (73.0)	14216 (75.2)	
Yes	5504 (25.1)	814 (27.0)	4690 (24.8)	
**Drinking status**				<0.001
No	17362 (79.2)	1382 (45.9)	15980 (84.5)	
Yes	4554 (20.8)	1628 (54.1)	2926 (15.5)	
**Negative emotions**	Mean ± SD	Mean ± SD	Mean ± SD	
Perceived stress	6.55 ± 2.54	6.56 ± 2.52	6.55 ± 2.58	0.287
Depression	7.31 ± 6.23	7.98 ± 6.60	7.20 ± 6.61	<0.001
Anxiety	4.72 ± 4.64	5.09 ± 4.90	4.66 ± 4.60	0.002
**EQ-VAS**	73.42 ± 21.58	71.70 ± 23.08	73.69 ± 21.32	<0.001

EQ-VAS: European Quality-Visual Analogue Scale. RMB: 1000 Chinese Renminbi about US$140.

Among the participants, 3010 (13.7%) were smokers and 18906 (86.3%) were non-smokers. The smokers had a significant sex bias, with the majority being men (90.1%). The mean age of smokers was 44.29 years, which was higher than that of all participants (39.43 years) and non-smokers (38.65 years; p<0.001). Compared to non-smokers, smokers had a significantly lower education level (p<0.001), with more than half having a lower secondary education level or less. In addition, compared to non-smokers, smokers were more inclined to be retired/unemployed and belong to rural populations and low-to middle-income households. Additionally, smoking was often accompanied by alcohol consumption and a high percentage of chronic diseases (all p<0.05). In terms of NEs, smokers scored higher than non-smokers in all dimensions, with depression (p<0.001) and anxiety (p=0.002) being significantly different between the two groups. The mean EQ-VAS score for the total sample was 73.42, with smokers having a lower EQ-VAS score (71.70; p<0.001) than non-smokers (73.69). Overall, the health status of smokers differed from that of non-smokers.

### Relationships between smoking, NEs, and HRQOL: Correlation analysis

As smoking was a binary variable, the η coefficient was determined to analyze the correlations of smoking with HRQOL and NEs. Because HRQOL and NEs were continuous variables, Pearson’s correlation analysis was used.

Table 2 shows the correlation coefficients between smoking, NEs, and the EQ-VAS score of the HRQOL. Smoking was weakly associated with HRQOL (η=0.032). As shown in Table 1, non-smokers had a higher HRQOL score than smokers. Among the NE parameters, the scores for depression (η=0.043) and anxiety (η=0.032) were also related to smoking. Combined with the data in Table 1, these findings indicate that smoking is associated with higher levels of depression and anxiety than non-smoking. However, no significant association was found between perceived stress and smoking in the correlation analysis (η=0.002). In addition, perceived stress (r= -0.296, p<0.001), depression (r= -0.298, p<0.001), and anxiety (r= -0.291, p<0.001) were negatively associated with HRQOL scores.

**Table 2:** Correlation among variables of the study participants in China from June to August 2022 (N=21916)

*Variable*	*EQ-VAS*	*Perceived stress*	*Depression*	*Anxiety*	*Smoking*
**EQ-VAS**	1				
**Perceived stress**	-0.296***	1			
**Depression**	-0.298***	0.440***	1		
**Anxiety**	-0.291***	0.472***	0.822***	1	
**Smoking**	0.032***	0.002	0.043***	0.032***	1

EQ-VAS: European Quality-Visual Analogue Scale.

*** p<0.001.

### Results of the mediation effect analysis

This study followed a four-step procedure to examine the mediating role of NEs on the association between smoking and HRQOL. The following condition was required: significant association between: 1) smoking and HRQOL, 2) smoking and NEs; 3) NEs and HRQOL; and 4) a significant indirect path coefficient of smoking influencing HRQOL through NEs. Satisfaction of the latter condition can be determined using the bias-corrected perceptual bootstrap method. As there was no significant association between stress and smoking (Tables 1 and 2), in the mediation analysis, we only studied the effects of the NE parameters depression and anxiety on the relationship between smoking and HRQOL. Sex, age, education level, monthly household income, urban and rural residence, chronic diseases, and alcohol use were included as covariates in all analyses.

**Figure 2:**
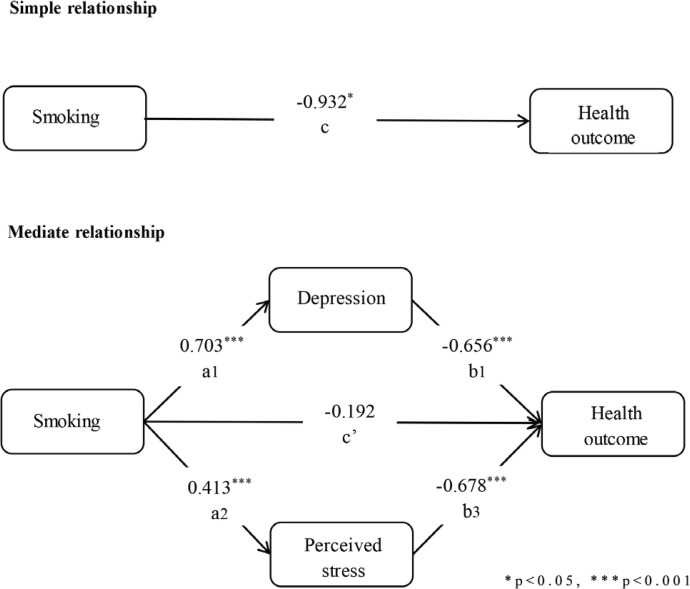
Path coefficients corresponding to unstandardized parameter estimates. *p<0.05, ***p<0.001T o

The settings of all regression models in the mediation analysis are shown in Table 3, and the path coefficients of the multiple mediation models are shown in Figure 2. The relative regression results showed that compared to not smoking, smoking had a negative effect on HRQOL (β= -0.932, p<0.001) and could significantly predict depression (β=0.703, p<0.001) and anxiety (β=0.413, p<0.001). When depression and anxiety were controlled for, smoking had no significant predictive effect on HRQOL compared with not smoking (β= -0.191, p=0.665), which means that more severe depression and anxiety are related to poor HRQOL. Likewise, when smoking status was controlled for, depression (β= -0.656, p<0.001) and anxiety (β= -0.678, p<0.001) had effects on HRQOL. These results showed that NEs play a mediating role in the influence of smoking on HRQOL.

**Table 3:** Regression coefficients, standard errors, and model summary information for the parallel multiple mediator model among the study participants in China (N=21916)

*Predictors*		*Y*		*M_1_*		*M_2_*		*Y*
		*β*		*β*		*β*		*β*
**X (Ref. non-smoking)**	c	-0.932*	a_1_	0.703***	a_2_	0.413***	c’	-0.192
**M1**							b_1_	-0.656***
**M2**							b_2_	-0.678***
**Constant**	i	78.036***	iM_1_	5.661***	iM_2_	3.544***	iY	84.150***
**Model fit**	R^2^	0.1003***		0.143***		0.129***		0.332***

* p<0.05,

*** p<0.001.

### Total, direct, and indirect effects of smoking on health

Table 4 summarizes the total, direct, and indirect effects of smoking on HRQOL through NEs (depression and anxiety). The 95% BCa CI of the overall total effect was [β= -0.932 (-1.846 − 0.019)], excluding 0; that is, smoking was associated with poor HRQOL. The bootstrap CI of the overall direct effect was [β= -0.192 (-1.058–0.675)], including 0, indicating that smoking had no direct influence on HRQOL after adjusting for other variables. The bootstrap CI of the overall mediating effect of NEs was [β= -0.740 (-1.062 – -0.427)], excluding 0; that is, smoking was related to poor HRQOL through a higher level of NE, with a mediating effect of 79.4%. Specifically, depression [β= -0.461 (-0.664 – -0.268)] and anxiety [β= -0.279 (-0.435 – -0.138)] had significant relative mediating effects on the influence of smoking on HRQOL, and they played a complete mediating role, with the relative mediating effects of depression and anxiety accounting for 49.5% and 29.9%, respectively.

**Table 4:** Total, total indirect, specific indirect, and direct effects of the multiple mediator model among the study participants in China (N=21916)

*Effect*	*Estimate*	*Boot S.E.*	*Boot (95% CI)*	*|a_i_b_i_/c|*
**Total**	-0.932	0.467	(-1.846 – -0.019)	
**Direct**	-0.192	0.442	(-1.058 – 0.675)	
**Total indirect**	-0.740	0.163	(-1.062 – -0.427)	79.4
**Specific indirect**				
Depression	-0.461	0.101	(-0.664 – -0.268)	49.5
Anxiety	-0.279	0.076	(-0.435 – -0.138)	29.9

All control variables were included. Boot S.E.: bootstrap standard error. Boot (95% CI): bootstrap 95% confidence interval; |*a_i_b_i_ /c*|: proportion of relative mediation effect.

## DISCUSSION

our knowledge, this is the first study focusing on psychological parameters involved in the relationship between smoking and HRQOL in the general Chinese population. In this national population-based study, we assessed the differences between smokers and non-smokers using descriptive statistics, and investigated the association between smoking and HRQOL, as well as the mediating role of NEs in this association. Smoking status, depression, anxiety, and HRQOL were correlated. Regression analysis showed that smoking was associated with higher depression and anxiety levels than not smoking, and higher depression and anxiety levels were associated with poorer HRQOL. The results of the mediating effect analysis indicated that smokers were affected more by anxiety and depression than non-smokers in terms of HRQOL. This verified hypothesis 1 that smoking is negatively correlated with HRQOL. Despite using different tools to measure HRQOL, previous surveys in different countries^16,26^ agreed that smoking was associated with poor HQROL. The HRQOL of Chinese smokers was significantly lower than that of the general population, similar to the results of a previous national survey^27^. This may be because smokers experience obstacles in mental health, thereby affecting their subjective assessment of HRQOL, whereas ex-smokers or never smokers are superior regarding emotional and mental health^28^. Therefore, our study focused on HRQOL differences between smokers and non-smokers in China and the psychological mechanisms influencing HRQOL, which is of great significance in strengthening the social attention to the health of smokers and improvement of HRQOL in smokers.

Similar to the findings of previous studies, smoking was associated with higher levels of depression and anxiety^7^. Because of long-term smoking, nicotine causes changes in neurotransmitters in the brain that affect mood^29^. Smoking can also lead to a disorder of the hypothalamus-pituitary-adrenal system and high levels of secreted cortisol, resulting in dysfunction of the neurobiological system, leading to depression and anxiety^30^. Although we also included perceived stress as an NE parameter in hypothesis 2, the results showed no significant correlation between smoking and stress. The relationship between smoking and stress remains controversial as some studies suggest that smoking is closely related to stress, whereas others suggest that there is no significant relationship between these factors^31^. Thus, the interaction mechanism between smoking and stress may be complex, and further longitudinal studies are needed to explore its causal relationship.

In the present study, depression and anxiety were associated with lower HRQOL. Previous studies in subgroups such as chronically ill^32^ and elderly^33^ populations have explained the association between mental disorders and low HRQOL, and these findings are consistent with the results of our current research. Our study provides new evidence for this relationship in a general population and in smokers in a national sample, and may help to better understand the psychological determinants of improving HRQOL.

Through bootstrap mediation analysis, we also partially verified hypothesis 2 proposed in this study that the NE factors depression and anxiety play an indirect role in the association between smoking and HRQOL. The results showed that smokers had higher levels of depression and anxiety than non-smokers, adversely affecting their HRQOL. Depression and anxiety, respectively, mediated 49.5% and 29.9% of the differences in smoking and health status. After adjusting for control variables and depression and anxiety scores, the direct link between smoking and HRQOL disappeared, indicating that depression and anxiety played a mediating role. This is similar to the findings reported by Milic et al.^16^ where, after adjusting for depression scores, the association between smoking and HRQOL disappeared. This emphasizes that depression and anxiety play key roles in the relationship between smoking and HRQOL. Smoking cessation measures can effectively improve mental health, and mental healthcare may reduce the incidence or prevalence of smoking. However, additional investigations are required to establish the temporal association between mental health and smoking.

### Limitations

This study has several limitations. First, it was based on cross-sectional data. The results can only demonstrate association but cannot establish a causal relationship. Prospective studies are needed to compare smoking status, NEs, and HRQOL. Second, smoking, stress, depression, anxiety, and HRQOL were measured by self-administered questionnaires, and the results may be affected by self-report and social expectation biases. Third, NEs mainly involve the study of depression, anxiety, and perceived stress, but there is a lack of awareness of other NEs, such as anger, loneliness, and panic. Future research should focus on including these emotions. Fourth, according to a question raised by Baron and Kenny^34^, the feedback effect between variables leads to simultaneity bias, that is, the two-way causal problem. For example, although we propose that smoking predicts occurrence of NEs, people smoke to reduce their NEs^35^. Finally, although we adjusted for control variables in the study, there may still be unmeasured confounding factors (such as lifestyle and demographic variables). Thus, it cannot be determined if the observed or unobserved effects are related to the measured variables or the unobserved factors.

### Implications

First, our results provide deeper insight into the relationship between smoking and HRQOL from a psychological perspective. From an academic point of view, we suggest that studies of substance abuse should be combined with those of mental health, which may help to provide additional insight for improving the health status of smokers. Second, future research needs to include large-scale longitudinal studies to determine the causal relationships between smoking, NEs, and HRQOL, to provide more information on internal connections. Third, from a practical point of view, we suggest that professionals, such as public health institutions, policymakers, and community personnel, pay more attention to the HRQOL of smokers and their psychological impact mechanism, to inform both smokers and non-smokers of the impact of smoking on HRQOL. In addition, when formulating health policies and carrying out smoking cessation campaigns, attention should be paid to smokers and people with high NE levels. Improvements in mental health and smoking prevention should be combined to create a healthier social environment. In addition, access to such information also has the potential to help smokers take proactive measures to improve their HRQOL.

## CONCLUSIONS

We found that HRQOL (EQ-VAS score) was significantly lower in smokers than in non-smokers in the Chinese population and that smoking was associated with higher NEs (depression and anxiety). According to the mediator effect analysis, depression and anxiety play key roles in the association between smoking and HRQOL. This suggests that smokers have higher levels of depression and anxiety than non-smokers, which leads to lower HRQOL. This finding reveals the relationship between NEs in smoking and HRQOL, emphasizes the role of mental health in smoking and HRQOL, and provides new perspectives for smoking cessation campaigns and HRQOL improvement for the Chinese smoking population.

## Data Availability

The data supporting this research are available from the authors on reasonable request.
